# Elevated Lp(a) and course of COVID-19: Is there a relationship?

**DOI:** 10.1371/journal.pone.0266814

**Published:** 2022-06-08

**Authors:** Agnieszka Pawlos, Paulina Gorzelak-Pabiś, Mateusz Staciwa, Marlena Broncel

**Affiliations:** Department of Internal Diseases and Clinical Pharmacology, Laboratory of Tissue Immunopharmacology, Medical University of Lodz, Lodz, Poland; Università degli Studi di Milano, ITALY

## Abstract

**Background:**

Lipoprotein (a)–Lp(a) has proinflammatory, prothrombotic and proatherogenic properties and may theoretically influence the course of COVID-19.

**Objectives:**

The aim of the study was to explore whether patients hospitalized due to COVID-19 with Lp(a) ≥30mg/dl may develop a worse course of the disease, increased incidence of thromboembolic complications, intubation and ICU hospitalization or death.

**Patients and methods:**

A retrospective analysis was performed of 124 patients hospitalized due to COVID-19 in the Department of Internal Diseases and Clinical Pharmacology between 29 November 2020 and 15 April 2021. The only exclusion criterion was age≥80 years. Patients were divided into two groups: 1. COVID-19 patients with Lp(a) <30mg/dl regarded as not elevated n = 80; 2. COVID-19 patients with Lp(a) ≥30 regarded as elevated n = 44.

**Results:**

A total of 124 COVID-19 patients were included in the study (66 men and 58 women) with a mean age of 62.8±11 years. COVID-19 patients with elevated Lp(a) level had significantly longer hospitalization time (11 vs. 9.5 days; p = 0.0362), more extensive radiological changes in CT scan (35 vs. 30%; p = 0.0301) and higher oxygen demand on admission (8 vs. 5L/min; p = 0.0428). Elevated Lp(a) was also associated with significantly higher OR for High Flow Nasal Oxygen Therapy (HFNOT) OR = 3.5 95%CI(1.2;8.9), p = 0.0140, Intubation and ICU OR = 4.1 95%CI(1.1;15.2) p = 0.0423, Death OR = 2.8 95%CI(0.9;8.5), p = 0.0409.

**Conclusions:**

Elevated Lp(a) might be one of the factors which contribute to a more severe course of COVID-19; however, further studies including larger groups of patients are needed.

## Introduction

COVID-19 has a varied course and may be particularly severe in patients with risk factors such as older age, cardiovascular diseases, diabetes and obesity [1, 2]. The severity of the disease is associated with elevated inflammatory markers including CRP, IL-6 and ferritin, which take part in the so-called ‘cytokine storm’ [3]. A hyperinflammatory response is observed in COVID-19 patients, which may contribute to multiorgan failure and a higher mortality rate [4]. SARS-CoV-2 infection is also associated with a prothrombotic state, and patients are prone to developing thromboembolic complications. A meta-analysis performed by Malas et al. shows that COVID-19 patients with thromboembolism have 74% higher mortality than those without (OR, 1.74; 95%CI, 1.01–2.98; p = 0.04) [5].

The reasons for the varied inflammatory response and developing thromboembolic complications in COVID-19 patients remain unclear. One of the possible factors contributing to increased inflammatory response, thromboembolic complications and mortality in COVID-19 patients may be Lipoprotein(a). Lp(a) is built up of a low-density lipoprotein particle with apoB (apolipoprotein B) and apoA (apolipoprotein[a]) [6]. Apo(a) has a similar structure to that of plasminogen, however, it has no fibrinolytic properties and it may impede clot lysis. Lp(a) appears to have a strong influence on atherosclerosis development due to its proinflammatory and prothrombotic properties. Lp(a) level is genetically determined and constant. The levels exceeding 30 or 50mg/dl are considered elevated and affect 20–30% of the global population [7]. Although no data exists on Lp(a) levels in patients with SARS-CoV-2 infection, there are strong theoretical grounds to suspect an association between Lp(a) and COVID-19. Elevated Lp(a) might be a potential risk factor of the hyperinflammatory response and thromboembolic complications in patients with COVID-19.

The aim of the study was to establish whether patients hospitalized due to COVID-19 with Lp(a)≥30mg/dl may develop a worse course of disease, increased incidence of thromboembolic complications, intubation and ICU hospitalization or death.

## Patients and methods

The study was conducted in the period between 29 November 2020 and 15 April 2021. It included 124 COVID-19 patients aged below 80 years who were hospitalized in the Department of Internal Diseases and Clinical Pharmacology, Bieganski Hospital, Lodz, Poland due to COVID-19. The study randomly included all COVID-19 patients who were hospitalized in the department between 29 November 2020 and 15 April 2021 (n = 150). All patients aged ≥80 years were excluded (n = 26), due to advanced age and multiple comorbidities that may distort the clinical picture. Therefore, 124 COVID-19 patients were included in the study.

These records (n = 124) were retrospectively analyzed for age, sex, height, weight, hospitalization time, lipid profile, Lp (a), statin intake, CRP, IL-6, procalcitonin, ferritin, fibrinogen, glucose, HbA1c, GFR, proteinuria, NT-proBNP, INR, thrombin time, prothrombin index, APTT, D-dimer, hsT, homocysteine, thromboembolic complications, atherosclerosis, morphology parameters, i.e. PLT, WBC, lymphocytes, neutrophils, admission saturation, discharge saturation and percentage of lung involvement assessed in CT by a qualified radiologist. All parameters were taken from a blood test panel performed on admission; however, some variables rose significantly during hospitalization and were frequently monitored (CRP, IL-6, D-Dimers), and in these cases, the highest values were recorded. For IL-6, it the highest value before tocilizumab administration was recorded.

Lp(a) was measured routinely in all hospitalized COVID-19 patients once on admission: Lp(a) level is genetically determined and remains stable during the lifetime [8]. Lp(a) measurement was performed by kinetic nephelometry (Immage Immunchemie System, Fa. Beckman Coulter). The patients were then divided into two groups based on the result: 1. COVID-19 patients with Lp(a)<30mg/dl, regarded as not elevated (n = 80); 2. COVID-19 patients with Lp(a)≥30mg/dl, regarded as elevated (n = 44). The Lp(a) threshold of 30mg/dl was taken from existing guidelines (where <30 is a goal value) and from literature [9–11].

The distribution of the data was verified with the Shapiro-Wilk test. Most variables had a non-normal distribution. The relationships between pairs of groups were tested with the Student’s t-test (normal distribution) and the Mann-Whitney U-test (non-normal distribution). The relationships between categorical variables was assessed with the use of the chi square test along with OR. All the analyses were performed with Prism 9.0.0 software. The outcome variables included HFNOT (High Flow Nasal Oxygen Therapy), intubation and in-hospital death.

The study was approved by the Bioethical Committee of the Medical University of Lodz, Poland; Consent number: RNN/143/21/KE. Since it was a retrospective study based on medical records, informed consent from the patients was not required. The Bioethical Committee approved the retrospective study without obtaining informed consent from the patients.

## Results

A total of 124 COVID-19 patients were included in the study; 66 men and 58 women with a mean age 62.8±11 years. The mean BMI of the patients was 30.53±7kg/m^2^. Mean hospitalization time was 10.5±4 days. Baseline characteristics of patients included in the study are featured in S1 Table. For further analyses, the patients were divided into two groups:

- Group 1: COVID-19 patients with Lp(a)<30mg/dl regarded as not elevated n = 80
- Group 2: COVID-19 patients with Lp(a)≥30 regarded as elevated n = 44

The characteristics of the study groups regarding Lp(a) levels were presented in (Table 1).

**Table 1:** Characteristics of the study group n = 124.

Parameter Median, 95%CI	Patients with Lp(a)<30 N = 80	Patients with Lp(a)≥30 N = 44	p
**Sex n female/n male**	37/43	21/23	0.99
**Age [years]**	64.5	66	0.3239
	95%CI (60; 67)	95%CI (64; 68)	
**BMI [kg/m** ^**2**^ **]**	29.05	29.75	0.7520
	95%CI (26.5; 34)	95%CI (24.6; 38.2)	
**Lp (a) [mg/dl]**	8	78.75	**<0.0001**
	95%CI (5.4; 11.2)	95%CI (60.8; 93.4)	
**Glucose [mg/dl]**	106.5	113.5	0.1263
	95%CI (104; 116)	95%CI (107; 137)	
**GFR [ml/min/1,73m2]**	78.65	72.03	0.3079
	95%CI (71; 86)	95%CI (66; 88.4)	
**Proteinuria [mg/dl]**	50	50	0.4626
	95%CI (10; 50)	95%CI (10; 50)	

### Atherosclerosis and lipid parameters

Out of 124 patients included in the study, 52 presented evidence of atherosclerosis, including prior diagnosis of ischemic heart disease, history of ACS or evidence of atherosclerotic lesions in CT scans. The incidence of atherosclerosis was significantly higher in patients with Lp(a)≥30mg/dl (54.5% vs. 35%; chi square test p = 0.0348); however, no significant differences were noted between the groups with regard to lipid profile, statins intake, homocysteine or Troponin T levels (Table 2).

**Table 2:** Lipid levels, homocysteine concentration, statin intake and incidence of atherosclerosis in COVID-19 patients associated with Lp(a) level.

Parameter Median, 95%CI	Patients with Lp(a)<30 n = 80	Patients with Lp(a)≥30 N = 44	P value
**% of patients with atherosclerosis**	28/80	24/44	**0.0348 (chi2)**
	35%	54.5%	
**hsTnT [ng/ml]**	0.012	0.015	0.2090
	95%CI (0.01; 0.021)	95%CI (0.01; 0.021)	
**TCH [mg/dl]**	157.5	154.5	0.3780
	95%CI (139; 166)	95%CI (132; 183)	
**LDL [mg/dl]**	81.5	84.5	0.3070
	95%CI (72; 94)	95%CI (72; 114)	
**HDL [mg/dl]**	39.8	42.85	0.1789
	95%CI (35.8; 42.3)	95%CI (35.9; 48)	
**Non-HDL [mg/dl]**	117	108	0.7005
	95%CI (97; 126)	95%CI (99; 136)	
**TG [mg/dl]**	145.5	138	0.5195
	95%CI (132; 155)	95%CI (116; 158)	
**% of patients taking statins during hospitalization**	29/80	18/44	0.6996
	36.25%	40.91%	
**Homocysteine [μmol/l]**	9.84	9.950	0.3857
	95%CI (9; 10.95)	95%CI (9.13; 13.41)	

### Course of COVID-19 associated with Lp(a) level

**Fig 1:**
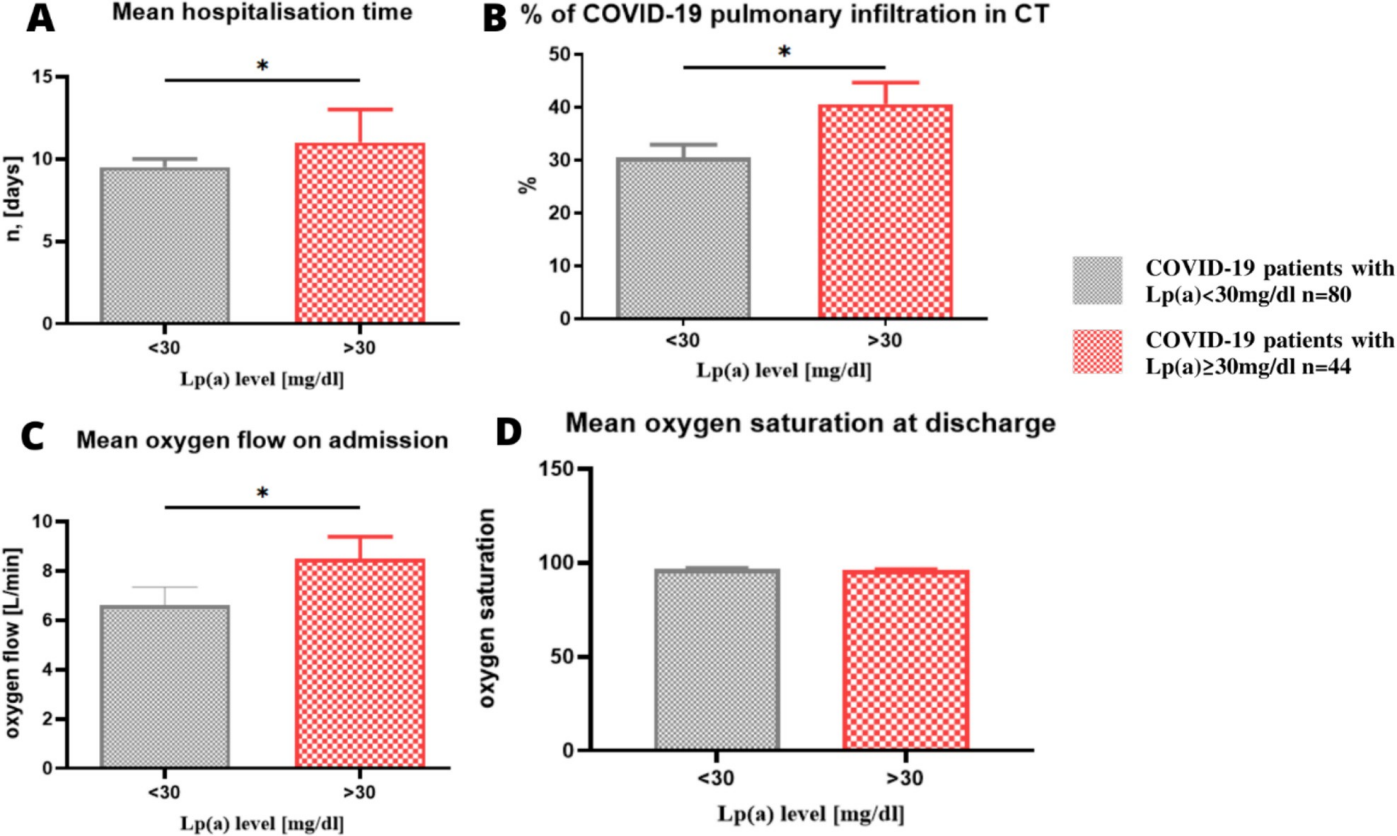
A. Hospitalization time in the COVID-19 patients with Lp(a)≥30mg—mean 11.52 days 95%CI (10;13) vs. 9.99 days 95%CI (9.1;10.9) in the patients with Lp(a)<30mg/dl one side p = 0.0362. B. Percentage of lung inflammatory infiltration in the COVID-19 patients with Lp(a) ≥30mg–mean 40.6% with 95%CI: (32.4;48.8) vs. 30.5% with 95%CI (25.7;35.3) in the patients with Lp(a)<30mg/dl one side p = 0.0301. C. Mean oxygen flow in the COVID-19 patients on admission to hospital 6.6L/min with 95%CI (5.1;8) in the patients with Lp(a)<30mg/dl vs. 8.5L/min 95%CI (6.6;10) one side p = 0.0428. D. Mean oxygen saturation in the COVID-19 patients at discharge from hospital did not differ significantly between the patients with Lp(a)<30mg/dl 96.7% with 95%CI (96.3;97.3) vs. 96.1% with 95%CI (95.3;97) p = 0.1505. All values on the graph are given in mean and SEM.

All the patients enrolled in the study required hospitalization due to COVID-19. The median length of hospital treatment was 10 days 95% CI (9;11), and it was significantly higher in the patients with elevated Lp(a) (median 11 vs. 9.5 days p = 0.0362) (Fig 1A). On admission, based on a lung CT scan, the percentage of COVID-19 inflammatory changes were assessed in each patient by a qualified radiologist. The degree of lung radiological changes ranged from 0 to 90% with a median of 30% 95%CI (25;35). The subjects with Lp(a) level ≥30mg/dl also developed more severe inflammatory changes in the lungs associated with COVID-19 (median 35% vs. 30% p = 0.0301) (Fig 1B). Out of 124 COVID-19 patients admitted to hospital, 103 (83.1%) required passive oxygen therapy. Minimal and maximal oxygen flow on admission was 0-17L/min with a median of 5L/min. The patients with elevated Lp(a) had a significantly higher oxygen demand on admission to hospital (median 8L/min vs. 5L/min in the patients with Lp(a)<30mg/dl p = 0.0428) (Fig 1C). Although the patients with elevated Lp(a) required longer hospitalization, more oxygen on admission and presented more COVID-19 changes in the lungs, their blood saturation at discharge was normal (median 97%) and it did not differ from oxygen saturation in the patients with Lp(a)<30mg/dl (median 97% p = 0.1505). The patients with Lp(a)<30mg/dl did not differ significantly from those with elevated Lp(a) in terms of inflammatory markers. i.e. CRP, IL-6, procalcitonin and ferritin. The median CRP level was 69.44mg/l 95%CI (51.6;87.6) vs. 81.05mg/l 95%CI (60.5;112.7), (p = 0.1559), respectively. The median IL-6 was 48.65pg/mL 95%CI (34.3;64.9) vs. 56.13pg/mL 95%CI (39.6;85) (p = 0.1892), respectively. The median procalcitonin was 0.095 95%CI (0.08;0.13) vs. 0.1 95%CI (0.09;0.2) (p = 0.0946) and the median ferritin 696 ng/ml 95%CI(537;930) vs. 860 ng/ml 95%CI (659;1574) (p = 0.1213).

For some of the patients, passive oxygen therapy up to 17L/min was not enough; in such cases, high-flow nasal oxygen therapy (HFNOT) was introduced with an oxygen flow up to 90L/min. HFNOT, intubation and ICU transfer and in-hospital death were considered as outcome variables (Table 3).

**Table 3:** Outcome variables of COVID-19 patients with regards to Lp(a) level.

Outcome	Patients with Lp(a)<30 n = 80	Patients with Lp(a)≥30 N = 44	P value Chi-square
**HFNOT**	7/80	11/44	**0.0140**
	8.75%	25%	
**Intubation**	3/80	6/44	**0.0423**
	3.75%	13.6%	
**In-hospital death**	5/80	7/44	**0.0409** *
	6.25%	15.9%	
**Composite negative outcome (HFNOT, intubation, in-hospital death)**	9/80	11/44	**0.0464**
	11.25%	25%	

* one-side p-value.

**Fig 2:**
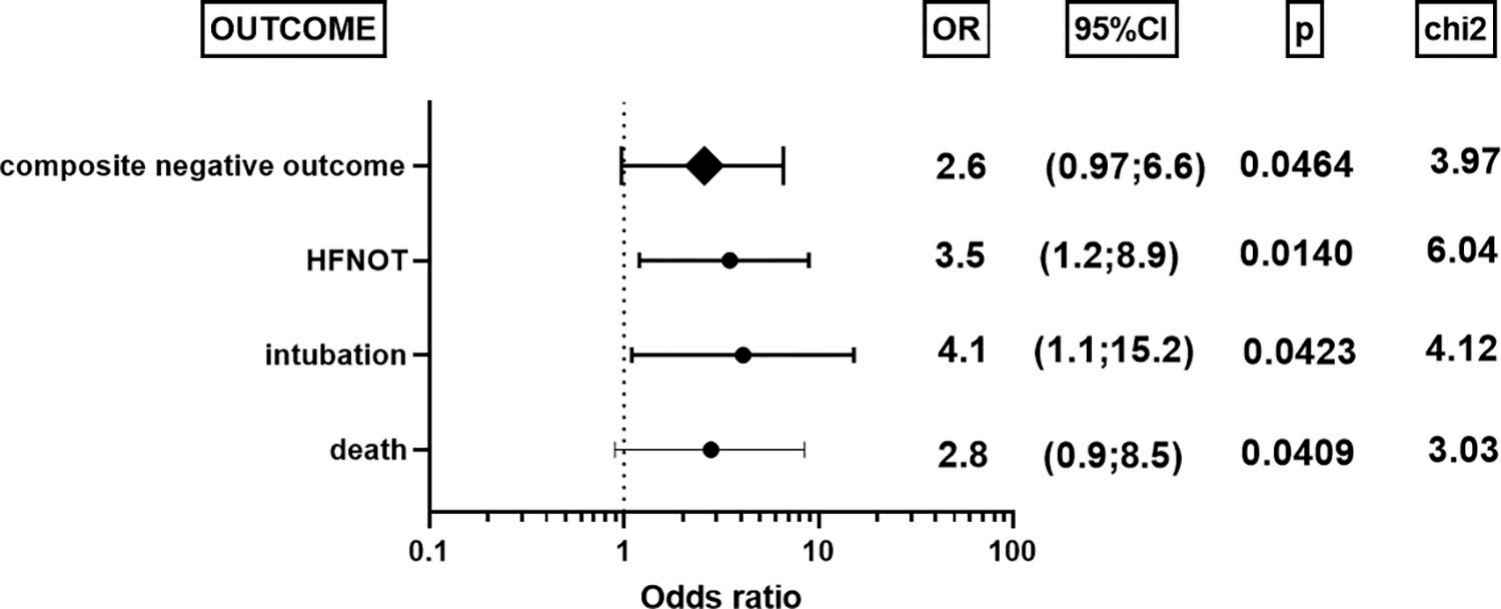
Odds ratio for the patients with Lp(a)≥30mg/dl /patients with Lp(a)<30mg/dl for HFNOT (High Flow Nasal Oxygen Therapy), intubation, in-hospital death.

The number of patients requiring HFNOT were 11/44 (25%) in the Lp(a)≥30mg/dl group compared to 7/80 (8.75%) in the Lp(a)<30mg/dl group (chi square test: p = 0.0140). Similarly, 13.64% of patients (6/44) in the high Lp(a) group were intubated and transferred to the Intensive Care Unit compared to 3.75% (3/80) in the low Lp(a) group (chi square test: p = 0.0423). The death rate was 15.9% (7/44) in the high Lp(a) group compared to 6.25% (5/80) in the low Lp(a) group (chi square test: p = 0.0409). The odds ratio (with 95% CI) and p-values for death, intubation and HFNOT for all patients, i.e. elevated and non-elevated Lp(a), are presented in Fig 2.

### Lp(a) level in COVID-19 patients–coagulogram and pulmonary embolism

The increased Lp(a) level was significantly associated with elevated D-Dimers, whereas in the analyzed case/group there were no differences in other coagulation parameters including fibrinogen, INR, thrombin time, prothrombin time and APTT (Table 4). Among the patients with non-elevated Lp(a), 6.25% (5/80) experienced pulmonary embolism while among those with elevated Lp(a) it was 13.64% (6/44) (chi square test: p = 0.1663). Odds ratio for pulmonary embolism was 2.37 95%CI (0.63;7.4).

**Table 4:** Median coagulation parameters and D-dimers in the COVID-19 patients with non-elevated vs. elevated Lp(a) level.

Parameter Median, 95%CI	Patients with Lp(a)<30 N = 80	Patients with Lp(a)≥30 N = 44	P
**Fibrinogen [mg/dl]**	548	531	0.4958
	95%CI (473; 578)	95%CI (443; 612)	
**INR**	1.086	1.065	0.4254
	96%CI (1.06; 1.14)	95%CI (1.04; 1.11)	
**Thrombin time [s]**	14.40	14.70	0.2856
	95%CI (13.8; 14.7)	95%CI (14.1; 15.3)	
**Prothrombin index [s]**	12.45	12.25	0.8473
	95%CI (12.2; 13.1)	95%CI (11.9; 12.8)	
**APTT [s]**	33.15	34.15	0.4543
	95%CI (31.7; 35.3)	95%CI (32.2; 37.3)	
**D-Dimers [μgFEU/L]**	1001	1265	**0.0208** *
	95%CI (775; 1188)	95%CI (893; 1884)	

* one-sided p value.

## Discussion

In our study we have found that patients with Lp(a)≥30mg/dl had significantly longer hospitalization time, more extensive pulmonary radiological changes, higher oxygen demand on admission, higher incidence of HFNOT, intubation and ICU hospitalization and in-hospital mortality.

Commonly-known risk factors for COVID-19 include older age, obesity, cardiovascular diseases and diabetes [2]. Although the role of Lp(a) in the course of SARS-CoV-2 infection remains unclear, it has been proposed that it may influence the course of the disease due to its proinflammatory, prothrombotic and proatherogenic properties [12]. The blood concentration of Lp(a) is genetically determined by the *LPA* gene locus and remains stable during the lifetime [13]. Some studies suggest that impaired kidney function may influence Lp(a) plasma level, this being one of the few non-genetic factors [14]; however, our present patients did not differ significantly with regard to GFR or proteinuria level (Table 1). The study groups were also homogeneous in terms of age and gender structure.

Lp(a) oxidized phospholipids (OxPLs) exert pro-inflammatory effects by causing inflammation in the arterial wall [15]. Our present findings indicate that patients with elevated Lp(a) generally presented higher serum concentrations of inflammatory markers such as hsCRP and IL-6; however, the difference was statistically insignificant. We hypothesize that the pro-inflammatory effect of Lp(a) on endothelial cells in COVID-19 patients may be local and connected with atherogenesis, and therefore may not be manifested in CRP or IL-6 serum concentration.

The inflammatory parameters in COVID-19 are significantly elevated: median hsCRP in our COVID-19 patients with Lp(a)≥30mg/dl was 81.05 with 95%CI (60.5;112.7). Meanwhile, in in 17 464 patients without SARS-CoV-2 infection after myocardial infarction, chronic inflammation underlying atherosclerosis was found to be associated with slight changes in hsCRP: the median hsCRP level was found to be 2.2 (IQR, 1.0–6.0) mg/L [16]. Therefore, in patients who experience a severe immunological response caused by SARS-CoV-2 infection, the pro-inflammatory effect of Lp(a) might not be noticeable at higher inflammatory marker concentrations.

Moreover, despite demonstrating no statistically-significant elevations in inflammatory markers, our patients with Lp(a)≥30mg/dl demonstrated a worse course of COVID-19, reflected as longer hospitalization time (11 vs. 9.5 days, p = 0.0362) more extensive pulmonary radiological changes (35% vs. 30%, p = 0.0301) and higher demand for oxygen on admission (8 vs. 5L/min, p = 0.0428). They were also more likely to demonstrate a significantly higher incidence of HFNOT (25% vs. 8.75%, p = 0.0140), intubation and OIT hospitalization (13.64% vs. 3.75%, p = 0.0423) and death (15.9% vs. 6.25%, p = 0.0409). The number of reports on this topic is very limited. Even so, Ruscica et al. report that Lp(a) level did not have significant prognostic value on in-hospital mortality in COVID-19 patients; however, median Lp(a) was 21nmol/L in that study and 17.2mg/dl = 43nmol/L in ours [17]. It is possible that the influence of Lp(a) on the course of COVID-19 was more noticeable in the present study due to its higher value. Our study suggests that Lp(a) might be an additional risk factor for a severe COVID-19 course, regardless of inflammatory markers. Nevertheless, further research involving larger groups of patients are required to confirm this theory.

SARS-CoV-2 infection is related to hypercoagulability and an increased incidence of thromboembolic events [5]. Theoretically, an elevated lipoprotein(a) level may also be associated with a higher risk of thrombosis. Apolipoprotein(a), which is a component of the Lp(a) particle, may impair fibrinolysis due to its structure being homologous with plasminogen, thus competing to bind with fibrin and inhibiting tissue plasminogen activator [8]. In our study, the Lp(a)≥30mg/dl group had significantly higher d-dimer concentration, (median 1001 vs. 1256 μg/l) p = 0.0208, with no difference in other coagulation parameters including fibrinogen, INR, thrombin time, prothrombin time and APTT (Table 3). Also, the incidence of pulmonary embolism was not significantly higher in the COVID-19 patients with elevated Lp(a), which is consistent with previous results obtained by Maio S Di et al. [18]. The prothrombotic properties of Lp(a) have been well confirmed in *in vitro* studies; however, a previous meta-analysis suggests that the association between elevated Lp(a) and VTE remains questionable [19, 20]. Moreover, in a genome-wide association study including 367 586 participants where Lp(a) concentration was predicted by the presence of mutations in the *LPA* gene, no significant relationship was noted between Lp(a) and VTE [21].

Another meta-analysis found coexisting cardiovascular diseases to be a risk factor for severe disease, death and fatal outcomes of COVID-19 [20]. In our study, the patients with Lp(a)≥30mg/dl had a significantly higher incidence of atherosclerosis (54.5% vs. 34% p = 0.0348), but did not differ significantly in terms of BMI, lipid parameters, homocysteine level or statin intake (Table 2). Lp(a) is said to be more atherogenic than LDL, due to additional presence of apo(a) which promotes atherothrombosis and facilitates preferable accumulation in the arterial wall [22]. The influence of elevated Lp(a) on the chance of IHD events is believed be greater in COVID-19 patients [18]. This may be crucial information for patients with Familial Hypercholesterolemia who often have elevated Lp(a) level, and therefore may be at even more increased risk of cardiovascular complications during COVID-19 [23]. Moreover, SARS-CoV-2 itself causes endothelial dysfunction, which may potentially result in accelerated development of atherosclerosis in COVID-19 survivors; in such cases, patients with additionally elevated Lp(a) might be particularly vulnerable to this effect [24].

## Conclusions

Elevated Lp(a) might contribute to longer hospitalization, more extensive pulmonary radiological changes, higher oxygen demand on admission, increased risk of high flow nasal oxygen therapy, intubation and ICU hospitalization and death. It may not be associated with any elevation in inflammatory markers or pulmonary embolism. However, further studies including larger groups of patients are needed to make such a conclusion.

### Limitations

The main limitation of the study seems to be its retrospective nature and small group sizes: wide confidence intervals make it difficult to precisely determine OR for HFNOT, intubation and death. The differences that we have noticed between COVID-19 patients with Lp(a)≥30mg/dl vs. <30mg/dl require further evaluation in a larger study.

## Supporting information

S1 Table (XLSX)

S2 Table Baseline characteristics of all the patients included in the study. (DOCX)
