# Supplementary material for: Elevated Lp(a) and course of COVID-19: Is there a relationship?
Source: PLoS One. 2022 Jun 8;17(6):e0266814. doi: 10.1371/journal.pone.0266814 (PMC9176856; doi:10.1371/journal.pone.0266814)
Supplement: S2 Table — (DOCX) [file pone.0266814.s002.docx]

| **Parameter**  **Median, 95%CI** | **COVID-19 patients**  **n=124** |
| --- | --- |
| **n men/n women** | 66/58 |
| **Age**  **[years]** | 65.5  95%CI (63;67) |
| **BMI**  **[ml/min/1,73m2]** | 29.05  95%CI (27.5;33) |
| **Hospitalization time**  **[days]** | 10  95%CI (9;11) |
| **Radiological changes in the lungs [%]** | 30  95%CI (30;40) |
| **Oxygen demand on admission**  **[L/min]** | 5  95%CI (5;8) |
| **Oxygen saturation at discharge**  **[%]** | 97%  95%CI (96;97) |
| **TCh**  **[mg/dl]** | 156  95%CI (144;166) |
| **LDL**  **[mg/dl]** | 84  95%CI (76;93) |
| **HDL**  **[mg/dl]** | 40.4  95%CI (37.3;43.3) |
| **Non-HDL**  **[mg/dl]** | 116  95%CI (104;126) |
| **TG**  **[mg/dl]** | 144.5  95%CI (129;155) |
| **Statins intake during hospitalization** | 47/124  37.9% |
| **Lp (a)**  **[mg/dl]** | 17.2  95%CI (13.1;23) |
| **IL-6**  **[pg/mL]** | 48.5  95%CI (37.9;60.5) |
| **CRP**  **[mg/l]** | 71.36  95%CI (60.5;90.2) |
| **PCT**  **[ng/ml]** | 0.1  95%CI (0.09;0.13) |
| **Ferritin**  **[ng/ml]** | 794  95%CI (617;920) |
| **Fibrinogen**  **[mg/dl]** | 544  95%CI (484;577) |
| **Glucose**  **[mg/dl]** | 109  95%CI (105;116) |
| **GFR**  **[ml/min/1,73m2]** | 76  95%CI (69;83) |
| **Proteinuria**  **[mg/dl]** | 50  95%CI (10;50) |
| **NT-proBNP**  **[pg/ml]** | 207.4  95%CI (167;270) |
| **INR** | 1.08  95%CI (1.05;1.11) |
| **Thrombin time**  **[s]** | 14.4  95%CI (14.1;14.7) |
| **Prothrombin index**  **[s]** | 12.4  95%CI (12.1;12.8) |
| **APTT**  **[s]** | 33.7  95%CI (32.2;35.3) |
| **D-dimers**  **[µgFEU/L]** | 1040  95%CI (878;1202) |
| **hsTnT**  **[ng/ml]** | 0.013  95%CI (0.011;0.015) |
| **Homocysteine**  **[µmol/l]** | 9.86  95%CI (9.2;10.9) |
| **The incidence of atherosclerostic lesions in CT scan** | 52/124  41.9% |
| **PLT**  **[n/µl]** | 206000  95%CI (183000;226000) |
| **WBC**  **[n/µl]** | 6400  95%CI (6000;7100) |
| **Lymphocytes**  **[n/µl]** | 800  95%CI (700;900) |
| **Neutrophils**  **[n/µl]** | 5150  95%CI (4300;5500) |
| **Pulmonary embolism** | 11/124  8.87% |
| **HFNOT** | 18/124  14.5% |
| **Intubation** | 9/124  7.3% |
| **Death** | 12/124  9.7% |

Table 1. Baseline characteristics of all the patients included in the study
